# 783. Knowledge of and attitude toward antibiotic use and resistance among medical students from a Thai public university

**DOI:** 10.1093/ofid/ofad500.844

**Published:** 2023-11-27

**Authors:** Porntita Sae-li, Sirashat Hanvivattanakul, Ruj Nana, Thana Khawcharoenporn

**Affiliations:** Faculty of Medicine Thammasat University, Bangkok, Pathum Thani, Thailand; Faculty of Medicine Thammasat University, Bangkok, Pathum Thani, Thailand; Faculty of Medicine Thammasat University, Bangkok, Pathum Thani, Thailand; Thammasat University, Bangkok, Krung Thep, Thailand

## Abstract

**Background:**

Assessment of knowledge of and attitude toward antibiotic use and resistance is essential for developing interventions that promote appropriate antibiotic use among medical students.

**Methods:**

An online survey study was conducted among preclinical- and clinical-year medical students of academic year 2022, at a Thai public university from January to April 2023. The knowledge score was calculated based on the correct responses to the 15 provided statements and attitudes were assessed by the 5 Likert scale.

**Results:**

Of the 313 participating students, 202 (65%) were in preclinical years, 111 (35%) were in clinical years, 44% were male, median grade point average was 3.5, and 71% reported history of internet searching for antibiotics. The median knowledge score about antibiotic use and resistance was higher among clinical-year students than preclinical-year students (12 vs. 9; p< 0.001). Less than 50% of preclinical-year students correctly responded to statements “There is no evidence of antibiotic resistance for gonorrhea” and “Antibiotic resistance can be transmitted from animals or humans to humans”, while less than 50% of both preclinical- and clinical years correctly responded to statements “Use of antibiotics in animals can lead to antibiotic resistance in humans”. Preclinical-year students were more likely than clinical-year students to agree that antibiotic resistance should be taught early in high schools and believe that we can always discover and develop new antibiotics, while clinical-year students were less-likely to agree that the Faculty of Medicine provided enough knowledge about antibiotic use and resistance during their study (P< 0.05). In multivariable linear regression analysis, factors associated with higher knowledge scores included higher academic year (P< 0.001), monthly household income ≥ $USD 3,000 (P=0.04), and high level of English proficiency (P=0.04).

**Conclusion:**

Provision of additional knowledge of and improving attitude toward antibiotic use and resistance were required and tailored based on the students’ academic years. The identified factors associated with knowledge score should be considered for implementing interventions to improve knowledge of and attitude toward antibiotic use and resistance.

**Disclosures:**

**All Authors**: No reported disclosures

